# Book Reviews

**Published:** 1892-12

**Authors:** 


THE USES OF WATER IN MODERN MEDICINE. By SIMON BARUCH, M. D., Attending Physician to the Manhattan General Hospital and New York Juvenile Asylum; Consulting Physician to the Montefiore Home for Chronic Invalids; formerly Chairman of the Board of Health of South Carolina, etc., etc. In two volumes. Physicians' Leisure Library. Detroit, Mich.: George S. Davis. 1892. Price, cloth, 50 cents; paper, 25 cents.

CEREBRO-SPINAL MENINGITIS. Its History, Diagnosis, Prognosis, and Treatment. By MARTIN W. BARR, M. D., Resident Physician in the Pennsylvania Training School for Feeble-Minded Children, Elwyn; formerly Resident Physician to the State Lunatic Hospital, Harrisburg, Pa. Physicians' Leisure Library. Detroit, Mich.: George S. Davis. 1892.

CONTRIBUTIONS OF PHYSICIANS TO THE ENGLISH AND AMERICAN LITERATURE. By ROBERT C. KENNER, A. M., M. D. Physicians' Leisure Library. Detroit, Mich.; George S. Davis. Price, cloth, 50 cents; paper, 25 cents. 1892.

GONORRHEA AND URETHRITIS. By G. FRANK LYDSTON, M. D., Professor of the Surgical Diseases of the Genito-Urinary System and Syphilology in the Chicago College of Physicians and Surgeons, etc., etc. Physicians' Leisure Library. Detroit, Mich.: George S. Davis. 1892. Paper, 25 cents; cloth, 50 cents.

These four books are among the latest outputs of the Physicians' Leisure Library series, that has become so popular and so useful. The first subject, Uses of Water in Modern Medicine, in two volumes, is handled most interestingly and instructively by Baruch. Few people pay sufficient attention to the use of water, either as a preventive, through its hygienic employment, or as a cure after disease or injury has occurred. There are many conditions of traumatism and disease that can be cured by water alone; or, to state it in another way, if we were limited to the employment of a single remedy, water would be chosen.

Cerebro-spinal meningitis is sometimes an obscure disease, especially in its approach, as the symptoms are so similar to many other neurotic disorders in the beginning; hence, it is important to watch carefully all the details of its manifestations and to be familiar with the literature that helps to interpret these manifestations. Barr's little monograph is excellent.

In his Contributions of Physicians to English and American Literature, Kenner has opened up a new channel of thought, and developed a new line of subjects for the Physicians' Leisure Library. To our mind, it would be better to invite contributions of this sort to the series, occasionally, rather than to confine it strictly to the technically scientific side of medicine. Kenner's little monograph will afford an entertaining and restful hour to many a weary doctor.

Gonorrhea and Urethritis is a subject that Dr. Lydston is competent to handle satisfactorily, from his large experience in the management of diseases of the genito-urinary tract. In this book of 216 pages, copiously indexed and moderately well illustrated, will be found many a useful hint to the busy doctor, who must necessarily be prepared to deal with these diseases, while the cheapness of the book ought to carry it into the office of every physician in the United States.

---

DISEASES OF THE STOMACH. By Dr. C. A. EWALD, Extraordinary Professor of Medicine at the University of Berlin; Director of the Augusta Hospital, etc. Authorized translation from the Second German edition, with special additions by the author. By Morris Manges, A. M., M. D., Attending Physician to Out-Door Department, Mount Sinai Hospital, New York, etc. With thirty illustrations; octavo, pp. xiv.—497. New York: D. Appleton & Co. 1892.

The importance of the knowledge of the diseases of the stomach, always great, is now urged upon the attention of the profession with more force than at any previous period in the history of medicine. Since physicians have become more enlightened as to the part defective nutrition plays in the cause or aggravation of almost all maladies that the human body suffers from, there has been considerable attempt made in all quarters where science prevails to pursue the studies of nutrition and its defects. The general practitioner must familiarize himself with the knowledge of the chemistry of the juices of digestion, as well as the diseases that the digestive organs are liable to take on. The specialist, too, must be well informed with reference to the intricacies of all questions relating to the digestive tract, else he will often fail in removing the local manifestation of disease to which his attention is especially and naturally directed. These thoughts have suggested themselves to us after an extended and careful reading of Ewald's treatise.

The volume is made up by grouping twelve lectures relating to diseases of the stomach. Lectures I. and II., comprising seventy pages, treat of methods of examination, and the determination of the acidity and acids of the contents of the stomach, together with the determination of the digestion of albumin, starch, absorption, motility, and the technique of the examination of the stomach. Lectures III. and IV. treat of stenosis and strictures of the cardiac and pyloric orifices, as well as megastria, gastrectasia, and dilatation of the stomach. Lecture V. deals with cancer of the stomach, and Lecture VI. with ulcer of the stomach. Lectures VII. and VIII. are taken up with the consideration of inflammations of the coat of the stomach, while Lectures IX., X., XI. deal with neuroses of the stomach. The title of the final lecture is Corelation of the Diseases of the Stomach to Those of Other Organs; the Practical Value of the Modern Chemical Tests.

While it is important to recognize functional digestive faults and to make haste to apply the proper methods to remove them, it is of the greatest concern that practical physicians should learn to diagnosticate the earliest symptoms of ulcers, cancers, and new growths in and about the stomach. We are certain that no one can read what Ewald says on these subjects without reaching the conclusion that he is a master of the subject, for he speaks with an authoritative knowledge that commands the respect of his readers, who readily become his students. We cannot give an analysis of this work in the short space allowed for its notice ; every interested physician must read the book for himself ; he will be a better physician after he has done so, provided he reads it understandingly, and follows its general teachings with intelligence and determination. The type and paper of the work make it easy to study even with eyes that are no longer young, and we anticipate the book will become one of the leading text-books in the schools on the subject of which it treats.

---

TRANSACTIONS OF THE EIGHTH ANNUAL MEETING OF THE AMERICAN CLIMATOLOGICAL ASSOCIATION, held at Washington, D. C., September 22, 23, 24, and 25, 1891. Octavo, pp. xvi.—276. Philadelphia: W. B. Saunders. 1892.

Though this Association has previously issued seven volumes, the one before us is the first that ever has come into the hands of the JOURNAL. We are glad to have so valuable an addition to our library shelves as this book affords. The eighth annual meeting was held in Washington, in September, 1891, and the papers contained in the volume have already been published, for the most part, in the journals. Whatever may be said of the value of journalistic literature, it is of vast importance that society transactions should be grouped in a single volume, where they can be readily referred to by the members and others interested. The work done by this Association at its eighth annual meeting was a credit to its members and an indication that the mission of the Association is being admirably fulfilled.

MEDICAL JOURNAL ADVERTISING. A Manual for Advertisers, edited by A. L. HUMMEL, M. D., Member of the American Medical Association, of the Association of Medical Editors, of the Mississippi Valley Medical Association, of the Philadelphia County Medical Society, and of the Alumni Association of the Medical Department, University of Maryland; formerly Business Manager of the *Medical Bulletin*, Publisher of the *Journal of Comparative Medicine and Surgery*, of the *University Medical Magazine*, and Managing Editor of the *Annals of Hygiene*. Published by Hummel & Parmele, Medical Journal Advertising Agents, 612 Drexel building, Philadelphia. 1892. 12mo., pp. 150. Price, $1.00.

Medical Journal Advertising is one of the most unique and handsome business books that has ever found its way to our table. It comes with the compliments of Hummel & Parmele, medical journal advertising agents, of Philadelphia, but the authorship of the book evidently abides with the editor, Dr. A. L. Hummel, whose exquisite taste and business skill is developed and accentuated throughout its pages. The first article, Medical Journal Advertising, by Dr. Hummel, furnishes most entertaining and delightful reading on a subject that we have heretofore considered uninteresting. Dr. Hummel infuses it with an interest that is surprising.

The several contributions to the pages, both from the medical and business sides of the subject, are also of a nature to bring interest and instruction to the reader. Dr. Love has discoursed happily upon the subject that he has chosen, namely, Medical Journal Advertising from the Standpoint of the Medical Journalist and the Busy Doctor; while Dr. Charles A. L. Reed has written two pages on Do Busy Doctors Read Medical Journal Advertisements? in which he answers the question in the affirmative in unmistakable language. Several of the well-known manufacturing chemists and pharmacists have also contributed short articles that go to make up an interesting whole.

In this book, also, we find a list of the medical journals of the United States, in which their circulation rating is given, obtained from four different sources. This table will prove useful to anybody desiring to ascertain the precise status of a medical journal with reference to its circulation. A carefully selected group of advertisements are added to the book, in which several medical journals tell their story to the public. The book is bound in white vellum, and printed upon heavy book paper, in beautiful type, making it altogether a gem.

---

THE PRINCIPLES AND PRACTICE OF BANDAGING. By GWILYM G. DAVIS, M. D., Universities of Pennsylvania and Gottingen. Member of the Royal College of Surgeons, England; Assistant Demonstrator of Surgery, University of Pennsylvania; Surgeon of the Out-patient Departments of the Episcopal and Children's Hospital; Assistant Surgeon to the Orthopedic Hospital. Detroit, Mich.: George S. Davis. 1891. Price, $3.00.

The art of bandaging must always be studied in the clinic to be appreciated and understood. Nevertheless, it is necessary that experts should give us well-written books on the subject, that are also intelligently illustrated. Dr. Gwilym Davis has met all the important indications of the subject in his treatise, and has presented it without superfluous sentences or elongated phrases. It is a work unique in its design and original in many of its parts. The illustrations were drawn by the author, and several of the bandages are original with him. He has taken from other treatises their most valuable points, and these, grouped with his own original work, serve to make up a most attractive volume,—one that students and younger surgeons will not be slow to possess.

---

ALL AROUND THE YEAR 1893. Entirely new design, in colors. By J. PAULINE SUNTER. Printed on heavy cardboard, gilt edges, with chain, tassels, and ring. Size, $4\frac{1}{2}$ x $5\frac{1}{2}$ inches. Boxed.

Mrs. Sunter's All Around the Year Calendar, for 1893, compares favorably with its predecessors, and is as graceful and pretty as anything she has done. The designs, done in several colors, are quaint and pretty. Sweet little lads and lassies issue forth, with just the right words, and combine to make a very charming love story. Seldom have we seen anything more pleasing than the twelve cards, each with its dainty design, which includes the calendar for the month. The designs are printed on cardboard, gilt edged, with rings, chain, cord, and tassels attached. Price, fifty cents. Lee & Shepard, publishers, Boston.

NAPHEY'S MODERN THERAPEUTICS. Medical and Surgical, including the Diseases of Women and Children. A Compendium of Recent Formula and Therapeutical Directions from the Practice of Eminent Contemporary Physicians, American and Foreign. Ninth edition, revised and enlarged. Vol. I. General Medicine and Diseases of Children, by ALLEN J. SMITH, M. D., Assistant Demonstrator of Morbid Anatomy and Pathological Histology, Lecturer on Urinology, etc., University of Pennslyvania, and J. Aubrey Davis, Assistant Demonstrator of Obstetrics, etc., University of Pennsylvania. Octavo, pp. xx.—1034. Philadelphia: P. Blakiston, Son & Company. Buffalo: Peter Paul & Brother.

The modest unpretending first edition of Naphey's Therapeutics, that appeared in 1871, is something in contrast to this formidable octavo volume of 1034 pages, bound in half-Russia and muslin with mottled edges. If a physician desires to obtain a concise statement of a special line of treatment, applicable to a particular disease, he will find it arranged in this book most admirably for reference, as well as satisfactory in its reading. It is rarely that one turns to any subject here considered without obtaining some useful hint in relation to it. The classification by anatomical systems is adhered to in the first part, beginning with the respiratory system and ending with toxic diseases. The second part considers the therapeutics of diseases of children, in which fevers and miasmatic diseases come first, then diseases of the respiratory tract, and, lastly, diseases of the alimentary tract. The type and paper are excellent, and this ninth edition has been carefully edited and systematically arranged. Unfortunately, it is sold by subscription only, hence cannot be obtained except through the publishers.

---

TRANSACTIONS OF THE ASSOCIATION OF AMERICAN PHYSICIANS. Seventh session, held at Washington, D. C., May 24, 25, and 26, 1892. Vol. VII. Philadelphia: Printed for the Association. 1892.

The seventh volume of the Transactions of this Association comprises the papers read at its annual meeting, held in Washington, in May, 1892. Dr. Victor C. Vaughan, in a paper of forty pages, gives an interesting bacteriological study of drinking water, that should be read by health officers of cities and towns, as well as others interested in the subject. There are many other important papers in the book, but one of the most practical for the general practitioner is that by Dr. Charles G. Stockton, entitled Misconceptions and Misnomers Revealed by Modern Gastric Research. This subject is one of great importance, hence we invite special attention to this excellent paper.

Transactions of the Thirteenth Annual Meeting of the American Laryngological Association, held in the City of Washington, D. C., September 22, 23, and 24, 1891. New York: D. Appleton & Company. 1892.

This volume is a report of the work of this famous Association for the year 1891. It is full of valuable literature by a number of the most eminent specialists in the department of laryngological medicine. Several of the papers have already appeared in the New York *Medical Journal*, but all interested physicians will find it needful to obtain the volume, in order to keep pace with the details of the work of the Association, many of which can be obtained in no other way.

chapter on The Differential Diagnosis of Functional and Organic Disease is one of the best in the book.

We do not hesitate in saying that it is one of the best books we have seen. It shows that it is the product of a well-ordered mind. J. W. P.

---

LEONARD'S PHYSICIANS' POCKET DAY-BOOK. Bound in red morocco, with flap, pocket, pencil, loop, and red edges. Price, postpaid, $1.00. Published by *The Illustrated Medical Journal Co.*, Detroit, Mich.

This handy day-book, which is also a vising list, is now in its fifteenth year of publication. The front part of it is occupied with dose tables and other useful pocket memoranda. It is good for thirteen months, from the first of any month in which it may be begun, and accommodates daily charges for fifty patients, besides having cash department and complete obstetric records. There are also columns for the diagnosis of disease, or for brief record of the treatment adopted, following each name-space. Name of patient needs to be written but three times in a month. The book is seven and one-half inches in length, and is three and one-half inches wide, so that it will carry bill-heads or currency bills without folding. It is bound in flexible covers, and weighs but five ounces, hence can be easily carried in the pocket.

---

OVER 1,000 PRESCRIPTIONS AND FAVORITE FORMULÆ, from Authors, Professors, and Practising Physicians. Cloth, 12mo, postpaid, $1.00. The Illustrated Medical Journal Co., Detroit, Mich.

The various formulæ contained in this volume are practical prescriptions of new and old remedies, for the various types of diseases that affect mankind. They are the favorite ones, of the various authorities, for the diseases indicated. The index is full and complete, thus rendering the whole book easy of access. The volume is copiously interleaved, so that on the blank pages can be recorded, by pasting or copying with pen or pencil, any other prescription suitable for any disease that is on the opposite page of the book; the complete index thus indexes each new formulæ one may see fit to copy into the pages of the volume. The whole is comprised in a handy cloth-bound volume of nearly 300 pages, and will be mailed to any address, upon receipt of its price, by the publishers.

